# Long-term debridement effect of denervated large sacrococcygeal pressure injury: A case report

**DOI:** 10.1097/MD.0000000000037012

**Published:** 2024-01-26

**Authors:** Yan Lu, Hai-Long Wu, Zong-Jian Luo

**Affiliations:** a Changchun University of Chinese Medicine, Changchun, Jilin Province, China; b Department of Orthopedics, The Affiliated Hospital to Changchun University of Chinese Medicine, Changchun, Jilin Province, China.

**Keywords:** case report, debridement, long term, paraplegia, pressure injury

## Abstract

**Rationale:**

Complicated pressure injury in paraplegic patients is common and difficult to manage. Previous case studies have documented short-term management; however, little is known regarding suitable approaches to long-term clearing of extensive pressure injury in the sacrococcygeal area under denervation.

**Patient concerns:**

A 53-year-old man was bedridden for 1.5 years owing to cervical vertebral fracture-dislocation (C5–C6), resulting in extensive sacrococcygeal pressure injury.

**Diagnoses:**

On admission, he presented with the injury complicated by infection (stage IV necrosis), and his vital signs were unstable.

**Interventions:**

The infection was treated with a range of antibiotics, including clindamycin phosphate, metronidazole, cefoperazone sodium, and sulbactam sodium. Debridement of the pressure injury was performed, helping remove the necrotic tissue and stimulate tissue regeneration.

**Outcomes:**

The patient was discharged after 88 days of hospitalization. The extent of the pressure injury at discharge was reduced compared with that at admission. At 4-month follow-up, the injury was nearly healed, with no signs of any further complications.

**Lessons:**

This case study suggests that wound debridement is a cost-effective and clinically efficacious approach to long-term complicated pressure injury management.

## 1. Introduction

Pressure injuries are common among patients that are bedridden over a long period. The prevalence of pressure injury is high in paralyzed patients, which has been reported as 33.9% in tetraplegic patients, 47.4% in paraplegic patients, and 9.6% in hemiplegic patients.^[1]^ Pressure injuries are usually accompanied by infection, which may be an independent predictor of mortality independently of other factors.^[2]^ Furthermore, pressure injuries are difficult to prevent and should be managed at the time of onset.^[3]^ Patients with infected pressure injuries require hospitalizations; however, hospital-based treatments are often limited by the lack of resources in developing countries. Moreover, treatments of paraplegic patients with pressure injuries remains challenging. Surgery, such as flap surgery, may be possible in some cases; however, patients with stage IV pressure injury, complicated by infection and tissue defects, are not eligible for this approach Long-term follow-up also remains paramount to good outcomes in patients with pressure injury. Nonetheless, little is known about long-term management of such cases. Herein, we report a case of long-term debridement performed for a patient with an extensive sacrococcygeal pressure injury under denervation. The presented details may support the management of similar cases.

## 2. Case presentation

A 53-year-old man was admitted to our hospital with severe pressure injury infection on July 26, 2018.

### 2.1. History of present illness

The patient had a car accident 1.5 years before admission, resulting in a fracture and dislocation of the cervical spine (C5–C6). He was treated surgically at a local hospital and remained bedridden over a long period. He gradually developed pressure injury in the lumbosacral area, chest, and back, which progressed over time owing to improper family care. Subsequently, 40 days before admission, he developed fever of 41.0 °C. He also experienced episodes of febrile convulsions.

### 2.2. History of past illness

The previous physical condition of the patient was normal. The patient had a history of smoking nearly 60 cigarettes per day for 35 years before the injury. He also had a history of drinking approximately 1 or 2 alcoholic drinks per day for 30 years before the injury. In addition, he reported experiencing morning cervical stiffness for 17 years and undergoing hospitalization for 2 years before this admission. The patient was diagnosed with ankylosing spondylitis and treated with methotrexate (the specific brand and dosage unknown), which resulted in no improvement. The patient had no history of hypertension. His blood pressure was low in the previous year, reaching 57/35 mm Hg; at admission, it was 84/56 mm Hg. The patient had a 2-year history of hyperlipidemia. He self-prescribed Xuezhikang (the specific brand and dosage is unknown) to control hyperlipidemia but experienced no symptom improvement. He had also been diagnosed with diabetes 5 years before the admission, with post- and preprandial blood sugar levels up to 21.5 mmol/L and up to 14 mmol/L, respectively. He was treated with self-administered metformin (specific brand and dosage unknown) without any improvement. The patient had an extensive medical history caused by vehicle accidents: subarachnoid hemorrhage 2 years prior to admission; cervical spine fracture 1.5 years after fracture operation; thoracic, rib, and vertebral body fractures 1.5 years prior to admission; and intermuscular venous thrombosis of both lower extremities for 1.5 years prior to admission. Except for the fractures, which were treated surgically, the patient’s injuries and conditions were not appropriately treated. At the time of presentation, the patient had been quadriplegic for 2 years, having sustained a spinal cord injury 2 years prior, with sensory loss below the nipple area. The patient’s conditions were correlated with the occurrence of spinal cord injury and pressure injury. The patient had no relevant family history.

### 2.3. Physical examination

The patient’s temperature and blood pressure values at admission were 38.5 °C and 84/56 mm Hg, respectively. An extensive pressure injury was observed in the sacrococcygeal region, where the skin was black, ulcerated, and necrotic, on an area of 25 × 10 cm. The patient presented with skin sensory loss below the elbow joint in both upper limbs, bilateral shrug muscle strength grade IV, triceps muscle strength grade III+, finger flexor muscle strength grade 0, the sensory plane at the level of the nipple, sensorimotor loss below the level of the nipple, and weakened tendon reflexes and increased muscle tone in the limbs. Negative bilateral Hoffman sign and Babinski sign were observed.

### 2.4. Laboratory examinations

**Figure 1.**
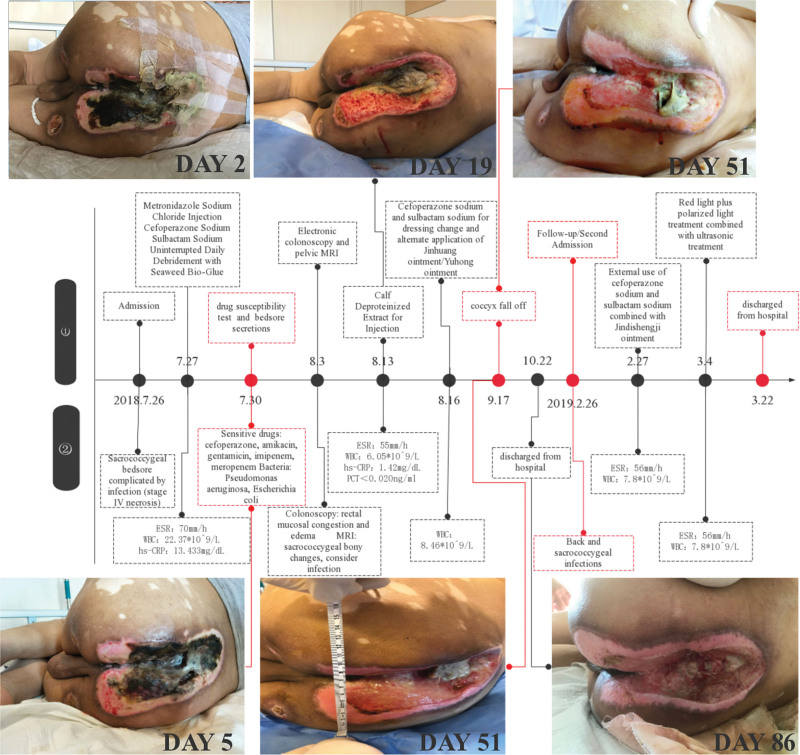
Timeline of the case.

**Figure 2.**
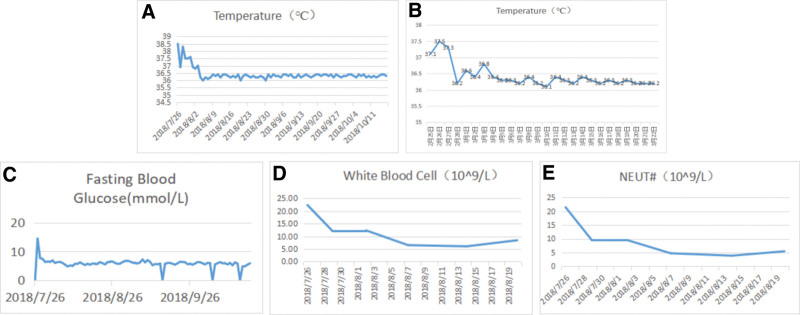
Clinical course of the patient. (A) The patient’s body temperature on the first admission was gradually stable on the seventh day after admission. (B) The patient’s body temperature on the second admission was gradually stable within a few days after admission. (C) On the first admission, symptomatic treatment was given, and fasting blood glucose gradually returned to normal on the 12th day. (D and E) On the first admission, the white blood cell count and neutrophil count gradually decreased and returned to normal on the 12th day.

At first admission, symptomatic treatment was administered (Fig. 1), and fasting blood glucose gradually returned to within normal levels on day 12. White blood cell and neutrophil counts gradually decreased and returned to within the normal levels on day 12 (Fig. 2). Laboratory test findings (complete blood count and erythrocyte sedimentation rate test completed at admission) and repeated measurement data (temperature, blood pressure, blood sugar levels, and blood routine) were recorded in detail for statistical analysis. Complete blood count values and blood sugar levels improved with the pressure ulcers after debridement.

### 2.5. Imaging examinations

Cervical spine computed tomography (performed at THE FIRST HOSPITAL OF JILIN UNIVERSITY, 2016-07-17, R04384212) scans revealed a fracture of the fifth cervical vertebral body and accessories. A magnetic resonance imaging scan of the lumbosacral spine revealed lumbar subcutaneous fat layer injury or infection. Furthermore, colonoscopy revealed rectal mucosal congestion and edema; the network structure disappeared, and the rectum and anus were not included in the lesions. Pelvic magnetic resonance imaging scans revealed abnormal signals in the soft tissue of the lumbosacral coccyx and pelvic floor muscles and changes in the sacrococcygeal bone, indicative of an infection.

### 2.6. Diagnosis and treatment

The patient was diagnosed with a sacrococcygeal pressure injury complicated with an infection (stage IV necrosis).

Based on examination findings, the pressure injury secretions were further examined. Drug susceptibility testing was performed to guide medication selection. The cost of hospitalization was then considered, and daily debridement treatment was performed. The patient’s vital signs was unstable at admission and anti-infection and symptomatic treatments were administered as soon as possible. Continuous low-flow oxygen was administered, alongside electrocardiogram-based, blood pressure, and blood oxygen level monitoring. A pressure-redistributing bed was also used to prevent further aggravation of the pressure injury. Clindamycin phosphate was administered intravenously at a dose of 1.8 g twice daily to control the infection. Invert sugar electrolyte at a dose of 1000 mL was administered intravenously for rehydration. The patient also received a single 500 mL intravenous injection of 5% glucose, and a single multivitamin injection (2 vials of injectable 12-compound vitamins + alanyl glutamine injection 20 g + compound amino acid injection 18AA 12.5 g insulin injection 8 u potassium chloride deca injection 1.0 g) was intravenously administered to supplement vitamin, amino acid, and other symptomatic rehydration. Dopamine hydrochloride injection at a dose of 40 mg was administered for symptomatic treatment to prevent endotoxin sepsis. On the second day of admission, clindamycin phosphate injections were discontinued due to deterioration of the patient’s condition. Cefoperazone sodium and sulbactam sodium 3 g were injected intravenously twice daily, combined with 500 mg of metronidazole sodium chloride intravenous injection twice daily to treat the infection.

The patient’s blood sugar levels were not well-controlled before admission, which was detrimental to pressure injury healing. We administered an intensive insulin pump therapy (insulin aspart injection) with a basal dose of 12 u: 4 u before breakfast, lunch, and dinner, administered before each meal.

Moreover, the debridement of the pressure injury was started that day. The treatment was as follows: the patient lay on the right side, and the pressure injury was fully exposed. The contaminated wound surface was disinfected, and the necrotic tissue was cleared from the periphery to the central area of the pressure injury until fresh granulation tissue was visible. After rinsing with a considerable amount of saline, sterile dressing was applied, completing the procedure. On admission day 3, the patient developed a fever, with a body temperature of 38.3 °C. A 2-mL aminobarbital injection was then injected intramuscularly to reduce the fever. Dehydration was treated with intravenous injection of 1500 mL of compound sodium chloride, 2 vials of 12-compound multivitamins for injection, and 25-compound amino quilt injection. Physical cooling treatment was performed to help relieve high fever symptoms. However, the patient’s fever persisted; thus, 1.0 g of arginine aspirin was administered intravenously once while continuing physical cooling. Owing to profuse sweating, 500 mL of 0.9% sodium chloride was administered intravenously for symptomatic rehydration. Ultrasound treatment was given to the patient, twice a day, for 75 minutes each time, to facilitate the recovery and rehabilitation of the spinal cord injury. Red light and polarized light were provided to the buttocks to facilitate the growth of granulation tissue at the site of the pressure injury and disintegrate and regenerate the muscles. The patient developed fever at the early stage of debridement. Special attention should be paid to the debridement at the later stage because excessive debridement in the initial stage may result in toxins being released into the blood. On day 9 of admission, the foul odor was slightly reduced, and removal of the necrotic tissue was continued. A considerable amount of yellow viscous and thick secretions was identified and removed. After rinsing with hydrogen peroxide and normal saline, the surface of the pressure injury was rinsed with metronidazole and treated with a sodium chloride injection. On day 16 of admission, a subcutaneous injection of insulin glargine was administered at a dose of 6 u at 8 pm, followed by insulin aspart injection (special charge) at a dose of 4 u, and subcutaneous injection before the 3 meals. On day 19 of admission, 1.2 g of deproteinized calf blood extract was injected by intravenous drip once a day to improve microcirculation, facilitate blood circulation at the site of pressure injury, and support wound healing. On day 50 of admission, the patient was provided a medical Vaseline dressing to cover the pressure injury in the right scapular region to facilitate healing. Cefoperazone and sulbactam sodium injection were discontinued during the dressing change to prevent drug resistance. Moreover, we tested pressure injury secretions and performed drug susceptibility tests. Topical antibiotics were cautiously considered, given the risk of drug resistance. On day 53 of admission, the soft tissue of the coccyx was severely corroded during the dressing change, and the coccyx was gray-black in color with only a small amount of necrotic fibrous tissue. On day 82 of admission, fresh wounds were identified after debridement. Fresh blood was exuding, and the necrotic tissue was absent; thus, the debridement of skin injury was discontinued. On day 88 of admission, the area of the pressure injury was narrowed, the stench was reduced, and the necrotic tissue was absent. In addition, the yellow viscous and thick secretions at the pressure injury site were reduced, and fresh blood was observed. The patient was stable and discharged (Fig. 1).

### 2.7. Outcome and follow-up

On February 25, 2019, the patient returned to our hospital. Physical examination showed that the size of the sacrococcygeal pressure injury was reduced from 25 × 10 cm to 4 × 2 cm, and no significant change in body sensation or muscle strength was observed. The size of the patient’s sacrococcygeal pressure injury was considerably reduced compared with that at the index admission, and the pressure injury on the buttocks also disappeared. On day 2 of this admission, debridement of the pressure injury site was performed, as previously described. The patient was injected cefoperazone sodium and sulbactam sodium at 3.0 g intravenously twice a day, combined with cefoperazone sodium and sulbactam sodium 3.0 g once a day for topical dressing change treatment. On day 8 of admission, a small amount of local exudation and fresh granulation tissue were identified at the pressure injury site during dressing change. In addition, the patient was treated with local red light and polarized light irradiation, combined with ultrasound treatment to facilitate the growth of fresh tissue. On day 10, to prevent the occurrence of local drug resistance, the topical administrations of cefoperazone sodium and sulbactam sodium were discontinued. The patient was administered vancomycin hydrochloride at a dose of 0.5 g during topical dressing change. He was more energetic than he was during the previous admission, and he showed confidence in the treatment and trust in his medical team.

## 3. Discussion

In this case, long-term debridement was used to treat a large-area pressure injury. This approach is cost-effective and clinically efficacious for patients with paraplegia complicated by large-area pressure injury (severity stage IV and above); thus, it can be popularized in community hospitals and clinics. However, this study was a case report, and the findings may have limited generalizability. Furthermore, little is known regarding the relevant debridement mechanisms. Debridement is also time-consuming, labor-intensive, and requires patient consent. Pressure injuries should be prevented and treated immediately after onset; however, resource availability may restrict the applicability of this approach. Nevertheless, debridement has some limitations, including but not limited to patient condition and expectations.

We also performed several treatments for the patient in this case report, including fluid therapy, antimicrobial treatment, treatment for underlying diseases such as diabetes, and ultrasound. Regarding the administered parenteral solutions, the electrolyte, vitamin, amino acid, insulin mixed solution has been shown to exhibit certain compatibility and stability.^[4,5]^ Fluid requirements typically increase when fever is considered; thus, the patients was administered symptomatic fluids following the prescription we described earlier.^[6]^ For ultrasound treatment, its use for pressure injury has been reported in the past,^[7]^ in which ultrasound was used alongside standard treatment to facilitate the healing of pressure injury.^[8]^ The ultrasound machine produces waves that transfer energy to the surface of the human body, targeting soft tissue deep to the epidermis. The energy from the ultrasound may affect deep tissues without causing harm to more superficial tissues.^[9]^ Regarding antimicrobial treatment, we mainly administered systemic drugs, although topical antibiotics were also considered. In some cases, topical antibiotics are useful alternatives to oral and parenteral drugs and offer some advantages such as ease of use, lower side effects, higher drug concentrations in infected areas, lower risk of developing bacterial resistance, and lower cost). Topical antimicrobial therapy aims to control microbial colonization, thus preventing the development of invasive infections; preventing and treating wound infections, pyoderma, burn infections and acne vulgaris; and eradicating *S. aureus* nasal carriage.^[10–12]^ In addition, there have been numerous reports on the treatment of anaerobically infected pressure injury with topical metronidazole and gentamicin.^[13–17]^

Pressure injuries are common worldwide. Their prevalence has decreased from 17.0% to 11.4%, according to some reports, owing to increased awareness and use of preventive hardware such as pressure-relief mattresses, skateboards, heel protection, as well as repositioning programs.^[18]^ However, in Sweden, where medical care is advanced, 1 in 10 hospital patients develops a pressure injury, suggesting that these types of complications are difficult to prevent.^[3]^ From 1990 to 2019, the age-standardized prevalence, morbidity, and annual mortality rates associated with pressure injuries decreased, with significant regional differences.^[18]^ Patient age and quality of home-based care may affect their risk of a pressure injury, suggesting a high need for nursing care. Although surgery may be a suitable approach to some cases of pressure injuries, the recurrence risk is high without proper home-based care owing to social and psychological risk factors and nursing care quality.^[19]^ Flap surgery can improve patient quality of life and alleviate pain. However, the risks of suture dehiscence, graft failure, and recurrent complications remain,^[20]^ despite improvements in surgical technique. Healing time is negatively correlated with the amount of microcirculatory perfusion at the center of the pressure injury,^[21]^ further suggesting the vital role of nursing staff in the care of patients who cannot turn over or even move their limbs without the help of others. Accordingly, the recovery of patients with pressure injury requires suitable on-going care that supports the strengthening of microcirculation perfusion at the center of the injury. In this case report, the patient’s injury improved with our approach, nearing full recovery, despite some on-going pain. Early treatment of pressure injuries is beneficial to patients and healthcare systems. The prevention and early treatment of pressure injuries improves patient survival.^[22]^ This report may support the management of chronic pressure injuries.

There are numerous causes of pressure injury, and the pressure on and compression of soft tissue play a critical role in the onset and progression of pressure injury.^[23]^ Among hospitalized patients worldwide, the pooled prevalence of pressure injuries was estimated as 12.8% for patients with grade ≥ 1 and 8.0% for patients with grade ≥ 2 injuries.^[24]^ Pressure injuries are also associated with high socioeconomic burdens and lower quality of life for individuals and societies.^[25]^ Pressure injuries can be treated with different methods, although which method may be most suitable in which case remains unclear. Accordingly, methods are continuously developed to treat this injury. For example, topical sevoflurane has been shown as highly effective for treating pain in patients with pus discharge.^[26]^ However, although this approach reduces pain and improves patient quality of life, it does not treat the injury.

Herein, the pressure injury occurred in the sacrococcygeal and scapular regions, resulting from the loss of limb innervation, inability to move the limbs, and continuous compression of soft tissues. Pressure injury after spinal cord injury is prone to recurrence; the most common recurrence sites are the sacrococcygeal region (82.1%), ischium (20.5%), trochanteric ulcers (15.4%), and other sites (2.6%).^[27]^ Thus, further research is required to elucidate how pressure injuries can be prevented in such cases.^[28]^ Herein, we described a paraplegic patient who had developed a pressure injury due to prolonged bed rest. Our approach helped heal the wound and improve the patient’s quality of life. Nevertheless, such injuries should be prevented or treated promptly to improve patient outcomes.^[22]^ This case report may assist physicians managing similar presentations. From 1990 to 2019, the age-standardized prevalence, morbidity, and annual mortality of pressure injury decreased, with significant regional differences.^[18]^ Previous research has indicated that the healing time is negatively correlated with the amount of microcirculation perfusion at the center of the pressure injury.^[21]^ Nursing care is paramount for patients who cannot turn or move their limbs. Improving outcomes in this context involves providing suitable care and strengthening microcirculation perfusion at the center of pressure injury.

This study had some limitations. In this case, the management of stress distribution and urinary incontinence was relevant to the patient’s treatment. Given the cost of hospital treatment, the patient’s family was involved in his care. Pressure redistribution and urinary incontinence management were performed by the family who were suitably instructed. Consequently, there were no records of these processes. Nevertheless, the patient’s medical team and family eventually cooperated, resulting in patient discharge and satisfactory outcomes. This cooperation may help ensure good long-term outcomes post-discharge.

### 3.1. Patient perspective

The medical team spent considerable time and energy on debridement every day, giving me confidence that the injury could heal despite its extent. Despite fecal incontinence experienced during the debridement process, which made the treatment difficult to complete, the medical team’s attitude help put me at ease. My mental state improved over time. As the treatment was daily and on-going, it helped ensure compliance, as evidenced by the readmission review. The treatment resulted in improved physical and mental state.

## 4. Conclusions

The existing treatment methods for paraplegia complicated with a pressure injury are limited. Herein, continuous debridement was performed, providing evidence that this method can be clinically efficacious and cost-effective for patients with paraplegia complicated with large-area pressure injury (severity stage IV and above). Pressure injuries are a type of chronic disease, which requires long-term treatment compliance to achieve good outcomes. Extensive evidence base for the benefits of debridement may improve clinician and patient confidence in this treatment.

## Acknowledgments

Thanks to the Orthopedics Department of the Affiliated Hospital to Changchun University of Chinese Medicine for the support to this article. We also thank Editage (www.editage.com) for English language editing.

## Author contributions

**Conceptualization:** Hai-Long Wu.

**Writing – original draft:** Yan Lu, Hai-Long Wu.

**Writing – review & editing:** Zong-Jian Luo.
